# Supplementary material for: Promoting arm movement practice with a novel wheelchair armrest early after stroke: A randomized controlled trial
Source: ArXiv. 2025 Oct 2:arXiv:2510.01753v1. Preprint. [Version 1] (PMC12622192)
Supplement: Supplement 1 [file NIHPP2510.01753v1-supplement-1.pdf]

Supplementary Material

Table A Clinical outcome measures – participants with less severe arm impairment at baseline (UEFM > 21)

	Intervention (Boost, n = 8)	Control (Booklet, n = 5)	Difference (95% CI)	P-value [a]
	Mean (SD)	Mean (SD)		
**Upper-extremity Fugl-Meyer**				
Baseline	29.6 (6.3)	36.6 (3.7)	[-13.6, -0.4]	0.06 [a]
Post-intervention	45.5 (7.9)	44.4 (5.9)	[-8.2, 10.4]	0.80 [a]
3-month follow-up	54.1 (6.0)	56.0 (10.2)	[-19.4, 15.7]	0.78 [a]
Change in score PI-BL	15.9 (5.8)	7.8 (2.9)	[2.2, 13.9]	0.01 [a]
Change in score FU-BL	24.1 (8.2)	18.8 (7.8)	[-8.2, 19.0]	0.37 [a]
**Box and Blocks test** [b]				
Baseline	6.2 (7.3)	5.4 (6.5)	[-8.8, 10.5]	0.85 [a]
Post-intervention	15.5 (8.9)	14.2 (10.5)	[-13.4, 16.0]	0.84 [a]
3-month follow-up	26.4 (9.0)	34.8 (12.2)	[-29.1, 12.5]	0.34 [a]
**Modified Ashworth Scale Wrist** [c]				
Baseline	0.9 (1.1)	1.2 (0.7)		0.49 [c]
Post-intervention	0.9 (0.9)	1.1 (0.7)		0.82 [c]
3-month follow-up	1.0 (0.9)	1.0 (1.2)		0.84 [c]
**Modified Ashworth Scale Elbow** [d]				
Baseline	1.7 (1.1)	1.7 (0.6)	[-1.1, 1.1]	0.98 [a]
Post-intervention	1.4 (1.2)	1.6 (1.1)	[-1.8, 1.4]	0.76 [a]
3-month follow-up	1.0 (1.6)	1.1 (1.1)		0.91 [c]
**Motor Activity Log** [e]				
How Well	2.7 (1.2)	3.2 (1.3)	[-2.8, 1.7]	0.57 [a]
Amount	2.9 (1.5)	3.7 (1.1)	[-2.9, 1.2]	0.37 [a]
**Shoulder Subluxation Distance Average (cm)**				
Baseline	0.6 (0.7)	0.0 (0.0)		0.09 [c]

Post-intervention	0.0 (0.1)	0.0 (0.0)	0.53 [c]
3-month follow-up	0.3 (0.5)	0.0 (0.0)	0.32 [c]
**Amount of pain no movement**			
Baseline	0.0 (0.0)	0.0 (0.0)	
Post-intervention	0.0 (0.0)	0.0 (0.0)	
3-month follow-up	0.0 (0.0)	0.0 (0.0)	
**Amount of pain with movement**			
Baseline	1.2 (1.6)	0.0 (0.0)	0.16 [c]
Post-intervention	1.4 (2.4)	0.0 (0.0)	0.29 [c]
3-month follow-up	1.3 (2.4)	1.5 (2.6)	1.00 [c]

SD = standard deviation; BL = baseline; PI = post-intervention; FU = 3 month follow-up

[a] Analyzed by Welch's t-test.

[b] Reported for paretic side.

[c] Analyzed by Mann-Whitney U test.

[d] Scores were either 1, 1^{+}, 2, 3, or 4, where 1^{+} was assigned the value of 1.5.

[e] Collected at 3-month follow-up.

Table B Clinical outcome measures – participants with less severe arm impairment at baseline (UEFM <= 21)

	Intervention (Boost, n = 9)	Control (Booklet, n = 9)	Difference (95% CI)	P-value [a]
	Mean (SD)	Mean (SD)		
**Upper-extremity Fugl-Meyer**				
Baseline	12.4 (2.7)	12.3 (6.0)	[-5.0, 5.2]	0.96 [a]
Post-intervention	20.7 (7.5)	18.7 (8.1)	[-6.3, 10.3]	0.62 [a]
3-month follow-up	26.8 (12.6)	30.6 (7.8)	[-15.6, 8.0]	0.50 [a]
Change in score PI-BL	8.2 (5.5)	6.3 (8.3)	[-5.6, 9.4]	0.60 [a]
Change in score FU-BL	14.3 (11.6)	19.4 (10.2)	[-17.6, 7.5]	0.40 [a]
**Box and Blocks test** [b]				
Baseline	0.4 (1.3)	0.0 (0.0)		0.37 [c]
Post-intervention	1.7 (2.5)	2.6 (5.7)		0.78 [c]
3-month follow-up	7.9 (12.2)	15.6 (11.4)		0.26 [c]
**Modified Ashworth Scale Wrist** [c]				
Baseline	0.9 (0.7)	0.9 (1.3)		0.67 [c]
Post-intervention	1.7 (1.2)	1.0 (0.9)	[-0.4, 1.8]	0.22 [a]
3-month follow-up	1.9 (1.1)	1.6 (0.9)		0.36 [c]
**Modified Ashworth Scale Elbow** [d]				
Baseline	1.7 (1.1)	1.7 (1.2)		1.00 [c]
Post-intervention	2.1 (1.3)	1.4 (1.1)	[-0.6, 1.9]	0.27[a]
3-month follow-up	2.6 (1.1)	2.3 (0.8)		0.36 [c]
**Motor Activity Log** [e]				
How Well	1.3 (0.8)	1.3 (0.8)	[-1.0, 1.0]	1.00 [a]

Amount	1.2 (0.8)	1.2 (1.0)	[-1.0, 1.1]	0.90 [a]
**Shoulder Subluxation Distance Average (cm)**				
Baseline	0.7 (0.6)	1.0 (0.9)	[-1.2, 0.5]	0.41 [a]
Post-intervention	0.8 (1.0)	0.3 (0.4)		0.33 [c]
3-month follow-up	0.7 (0.9)	0.5 (0.7)		1.00 [c]
**Amount of pain no movement**				
Baseline	0.8 (2.2)	0.0 (0.0)		0.37 [c]
Post-intervention	0.8 (2.2)	0.0 (0.0)		0.37 [c]
3-month follow-up	1.4 (2.2)	1.1 (2.1)		0.85 [c]
**Amount of pain with movement**				
Baseline	1.6 (2.3)	2.3 (2.8)		0.58 [c]
Post-intervention	2.4 (3.5)	1.6 (2.3)		0.61 [c]
3-month follow-up	4.8 (4.0)	6.7 (1.5)	[-5.4, 1.5]	0.24 [a]

SD = standard deviation; BL = baseline; PI = post-intervention; FU = 3 month follow-up

[a] Analyzed by Welch's t-test.

[b] Reported for paretic side.

[c] Analyzed by Mann-Whitney U test.

[d] Scores were either 1, 1^+, 2, 3, or 4, where 1^+ was assigned the value of 1.5.

[e] Collected at 3-month follow-up.
